# The Investigation of the Seebeck Effect of the Poly(3,4-Ethylenedioxythiophene)-Tosylate with the Various Concentrations of an Oxidant

**DOI:** 10.3390/polym11010021

**Published:** 2018-12-24

**Authors:** Joon-Soo Kim, Woongsik Jang, Dong Hwan Wang

**Affiliations:** 1 Materials Research Laboratory (MRL), University of California, Santa Barbara, CA 93106, USA; hikimjoons@gmail.com; 2 School of Integrative Engineering, Chung-Ang University, 84 Heukseok-Ro, Dongjak-gu, Seoul 06974, Korea; dndtlr2@cau.ac.kr

**Keywords:** conjugated polymer, doping concentration, seebeck effect

## Abstract

Poly(3,4-ethylenedioxythiophene)-tosylate (PEDOT-Tos) can be synthesized through an in situ polymerization and doping process with iron(III) p-toluenesulfonate hexahydrate as an oxidant. Both the Seebeck coefficient and the electrical conductivity were modified by varying the concentration of the oxidant. We investigated the effects of varying the concentration of the oxidant on the particle sizes and doping (oxidation) levels of PEDOT-Tos for thermoelectric applications. We demonstrated that an increase in the oxidant enabled an expansion of the particle sizes and the doping levels of the PEDOT-Tos. The modification of the doping levels by the concentration of the oxidant can provide another approach for having an optimal power factor for thermoelectric applications. De-doping of PEDOTs by reduction agents has been generally investigated for changing its oxidation levels. In this study, we investigated the effect of the concentration of the oxidant of PEDOT-Tos on the oxidation levels, the electrical conductivities and the Seebeck coefficients. As loading the oxidant of PEDOT-Tos, the Seebeck coefficient was compromised, while the electrical conductivity increased.

## 1. Introduction

Thermoelectric (TE) materials can interconvert thermal and electrical energies, which makes them useful for various applications, including the capture of waste heat and temperature control devices. The most promising materials for these applications are inorganic materials, such as Bi_2_Te_3_-based alloys, skutterudites, and clathrates [1,2,3,4]. The strong phonon scattering in the complex crystal structures in these materials decreases the thermal conductivity (*κ*), while maintaining the electrical conductivity (*σ*), similar to the concept of “electron crystal and phonon glass” materials for high-performance TEs [5]. The Seebeck coefficient (*α*), *κ*, and *σ* determine the dimensionless figure of merit (ZT), which is defined as 

(1) $$\mathrm{ZT}=\frac{\alpha^{2}\sigma \cdot T}{\kappa }$$

A higher ZT implies the powerful ability of a material for the TE. Despite the advantageous electrical properties of inorganic materials, the vacuum or inert-atmosphere process requirements, toxicity, and high cost of the rare-earth elements have hindered their further developments [6]. Conjugated polymers have been suggested as a means to solve these problems [7,8,9,10,11,12,13]. Even though their ZT values are significantly lower than those of inorganic materials, conjugated polymers are attractive as their structures can be easily modified, enabling tunability of their physical and chemical properties. Current studies on TE conjugated polymers are mostly focused on varying the doping levels and optimizing the oxidation point [14,15]. The Seebeck coefficient can be defined as entropy per charge carrier; it is inversely proportional to the electrical conductivity as the increase in the doping level shifts the Fermi level close to the carrier transport level, increasing the number of charge carriers in the material. Therefore, optimization of the doping level to achieve the maximum ZT is the most important goal for the use of conjugated polymer TEs. However, a saturation doping level is observed in a conjugated polymer depending on the chemical species. It cannot be doped excessively; overloaded dopants cannot further increase the doping level of the conjugated polymer.

Poly(3,4-ethylenedioxythiophene) (PEDOT) is a well-studied *p*-type conjugated polymer used as an electrode layer in organic photovoltaic (OPV) cells and transparent electrodes for organic thin-film transistors and electroluminescent (EL) devices [16]. Neutral PEDOT can be oxidatively polymerized in the presence of poly(styrenesulfonate) (PSS), which supports the dispersion of insoluble PEDOT particles in an aqueous medium, which can be used to cast thin conducting films (PEDOT:PSS) [17]. However, the presence of PSS can disrupt the connectivity of the PEDOT domains in thin films, leading to variation in the electrical conductivity over several orders of magnitude (10^−5^ to 1 S·cm^−1^), depending on the ratio of PEDOT/PSS and processing conditions [18,19,20,21]. On the other hand, PEDOTs synthesized by small-sized anions such as tosylate, triflate have been suggested for multiple electrical applications due to high electrical performance [22,23,24,25].

Several studies have proposed the use of PEDOT derivatives for TE materials by controlling the doping levels. Chang et al. investigated the TE properties for different PSS contents of PEDOT-PSS [18]. They reported that the Seebeck coefficient can be manipulated by the PSS molar ratio with a fixed PEDOT chain length. Optimization of the doping level of PEDOT-tosylate (Tos) by exposure to a reducing agent was studied by Bubnova et al. [19]. Once the PEDOT-Tos was synthesized, it was de-doped by tetrakis(dimethylamino)ethylene (TDTAE) to attain a maximum value of the power factor. PEDOT synthesized by self-inhibiting function oxidants such as iron (III) dedecylbenzenesulfonate (FeDBSA_3_) has been also reported to control basicity during synthesis in order to achieve high conductivity, but they also used reduction agents to optimize the doping levels [26].

The thermopowers and electrical conductivities of PEDOT-derivative thin films have been investigated as a function of the doping level. Control of the doping of PEDOT was the main aim in these studies; dimethyl sulfoxide (DMSO) assisted the doping process [18]. De-doping by a reducing agent was also used to study changes in the doping levels of PEDOT [19].

In this study, we varied the oxidation levels of PEDOT-Tos through the concentrations of iron(III) p-toluenesulfonate hexahydrate as a oxidant. We demonstrated that the doping capacity (saturation level of doping) was expanded by increasing the concentration of the oxidant, not by the reduction agents. This would allow for the optimization of the TE performances of conjugated polymers by another simple approach.

## 2. Materials and Methods

### 2.1. Synthesis of PEDOT-Tos

We synthesized the PEDOT-Tos in the following way; 50 µL of 3,4-ethylenedioxythiophene (EDOT, Aldrich, Saint Louis, MO, USA) was added to a solution of iron(III) p-toluenesulfonate hexahydrate (Aldrich, Saint Louis, USA) with different concentrations (1, 10, 20, 40, 60, and 80 wt % with respect to *n*-butanol) in 1 mL of *n*-butanol. The prepared solutions were coated on a glass substrate by spin-coating at 1000 rpm for 30 s; in situ polymerization and doping were performed at 100 °C for 5 min. The PEDOT-Tos films were washed in *n*-butanol several times to remove residual iron species as by-products. Powders of PEDOT-Tos were prepared by the same synthesis method as above and centrifuged in *n*-butanol to remove by-products and solvent.

### 2.2. Characterizations

A standard four-point probe measurement was performed to measure the electrical conductivities of PEDOT-Tos thin films on glass with patterned Au electrodes (a thickness of 70 nm) on the top of the films. A channel length of 0.2 mm and a channel width of 1 mm were made for the four-point probe conductivity contacts [27]. The thermopower values of the thin films were measured at room temperature using the same samples of the electrical conductivity measurements. Thermoelectric cooler (TEC) stages and thermal paste were used to provide a heat gradient in the PEDOT-Tos films; the temperature difference between TECs was controlled by thermocouples, and the voltages induced by the heat gradients in the samples were measured. The Seebeck coefficients can be calculated using the ratios between Δ*V* and Δ*T* of the films between two thermally isolated TECs. ^13^C Solid-state nuclear magnetic resonance spectroscopy (Advance DSX 300 MHz NMR, Bruker) was used to analyze the structure of PEDOT-Tos: δ = 20, 65, 98, 108, 127, 138 and 153 ppm. Transmission electron microscopy (TEM) images were recorded with a FEI Tecnai G2 Sphera microscope. A small-angle X-ray scattering (SAXS) diffractometer (XENOCS FOX2D multilayer optics for SAXS) was used to measure the size distribution of the synthesized PEDOT-Tos. The oxidation levels of PEDOT that were estimated by a ratio of Tos/EDOT were determined by XPS (X-ray Photoelectron Spectroscopy, Kratos, Manchester, UK). The sizes of the particles were measured by DLS (Dynamic Light Scattering, DynaPro NanoStar^TM^, Wyatt Technology, Santa Barbara, CA, USA).

## 3. Results and Discussion

We investigated the relationship between the thermopower and the electrical conductivity as a function of the concentration of the oxidant of PEDOT with a tosylate counter-ion (PEDOT-Tos). The synthesis of PEDOT-Tos was performed by the oxidation polymerization of EDOT and the doping of neutral PEDOT by tosylate [28]. We split the amounts of iron(III)·tosylate as 1, 10, 20, 40, 60, and 80 wt % with respect to *n*-butanol, and EDOT was added to each prepared solution. We denoted the PEDOT-Tos samples with numbers corresponding to the concentrations used in the synthesis. Inhibitors such as pyridine were not applied in this work. Even though conventional oxidants are acidic and may cause uncontrollable polymerization, the concentration of an inhibitor could be another variation for the work. Unexpected side effects may occur which is not a main objective of the experiment. However, in order to create a weak basic atmosphere to control the polymerization reaction with such a high acidic anion, those inhibitors should be added in future studies [26]. This work simplified the variable as the concentration of the oxidant to acquire its effects on PEDOT.

**Figure 1:**
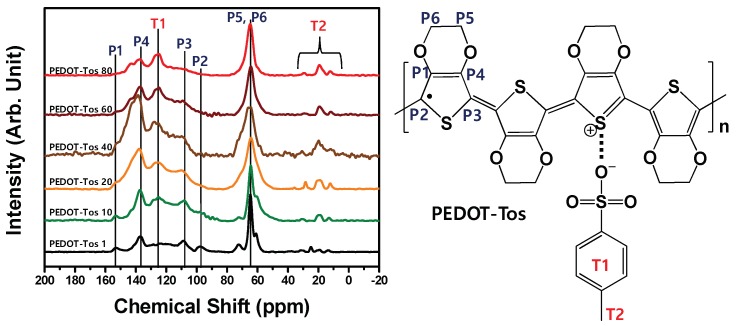
^13^C solid-state nuclear magnetic resonance (NMR) spectra of the Poly(3,4-ethylenedioxythiophene)-tosylate (PEDOT-Tos) samples are presented. The inset shows the chemical structure of PEDOT-Tos.

All PEDOT-Tos with changing the concentration of the oxidant were obtained by the conventional route, solution casting polymerization. In a similar way to Bubnova’s report, they were synthesized in sequence, and ^13^C solid-state NMR peaks in Figure 1 indicate the PEDOT-Tos samples were formed by each other with various concentrations of the oxidant. Unfortunately, solubility of the PEDOT is so poor that molecular weight analysis by GPC (Gel Permeation Chromatography) and other conventional soluble polymer analyses are not suitable. Especially, since iron (III) tosylate is known to generate crystal-like structures, there is a restriction in analysis methods. In this work, we found that target PEDOT-Tos samples were successfully formed by ^13^C solid-state NMR analysis. As observed in the spectra of PEDOT-Tos in Figure 1, the peaks around 20 ppm indicate the –CH_3_ group in the tosylate (counter-ion), while the peaks around 65 ppm represent the ethylenedioxy (–O–CH_2_–CH_2_–O–) group in the PEDOT chain [29,30,31,32]. The intensity (*I*_65ppm_) of carbons (P5, P6) in the ethylenedioxy group of the PEDOT chain and intensity (*I*_20ppm_) of carbons (T2) in the methyl group of the tosylate could present, roughly, the doping ratio of the PEDOT chain and the tosylate. However, the integration values are very rough which are hardly ever used as comparison data. The quantified oxidation levels are determined by XPS analysis. Additionally, their effects on the TE characteristics are dealt with.

**Figure 2:**
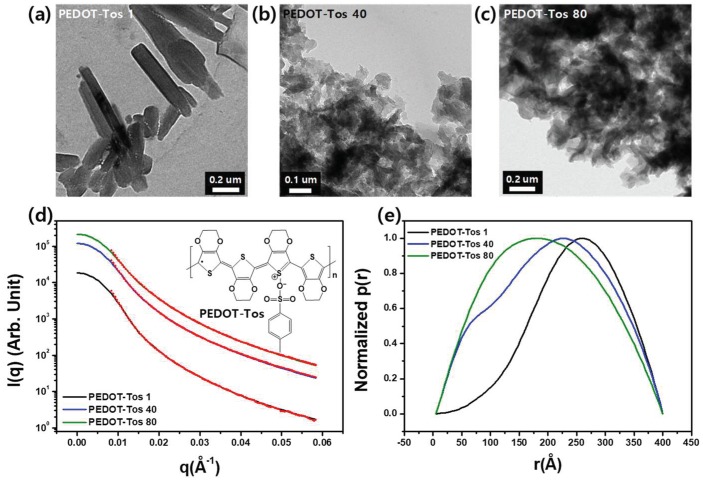
Transmission electron microscopy (TEM) images of the in situ polymerized and doped (**a**) PEDOT-Tos 1, (**b**) PEDOT-Tos 40, and (**c**) PEDOT-Tos 80 are presented. Additionally, (**d**) small-angle X-ray scattering (SAXS) analysis of PEDOT-Tos is also shown. The experimental intensities were plotted and fitted (red dotted curves) by the GNOM program. (**e**) Normalized *p*(*r*) was plotted by the GNOM program [33].

The electron microscopy measurements demonstrated that the morphology of aggregates of the synthesized PEDOT-Tos varied with the concentration of the oxidant. TEM images of the drop-cast samples showed significant changes in microstructure with large plate-like aggregates for PEDOT-Tos 1 (Figure 2a) and smaller aggregates for PEDOT-Tos 40 (Figure 2b) and PEDOT-Tos 80 (Figure 2c). The SAXS measurements at a wavelength of 1.54 Å in Figure 2d,e show systematic changes with the increase in the concentration of the tosylate. We attribute these differences to the particle sizes of the synthesized PEDOT-Tos. Aggregated large particles were formed with the increase in the concentration of the oxidant. The PEDOT-Tos powders were covered by Kapton films for the measurement. The intensity (*I*(*q*)) of scattering (*q* = 4π/*λ·*sin(θ/*2*)) is plotted in Figure 2d; the GNOM program was used to obtain the best-fit intensity. In addition, Figure 2e shows the probability (*p*(*r*)) of finding small particles at a distance of *r*. Both *I*(*q*) and *p*(*r*) indicate that the size of the aggregated PEDOT-Tos increases with the concentration of the oxidant. Additionally, DLS (Dynamic Light Scattering) was used to estimate the particle sizes of prepared PEDOT-Tos samples.

**Figure 3:**
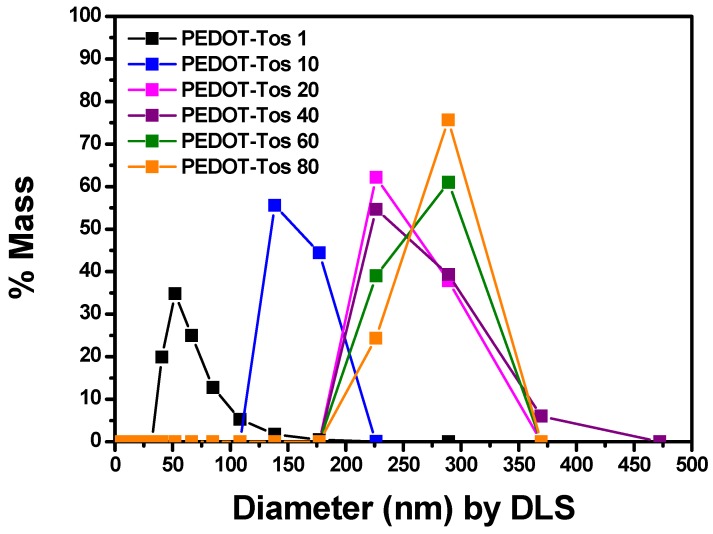
Particle sizes of the synthesized PEDOT-Tos depending on the concentrations of the oxidant are shown through dynamic light scattering (DLS) analysis.

We used a well-known DMSO (Dimethyl Sulfoxide), which is mainly used as a PEDOT solvent, to measure the relative particle size values. As shown in Figure 3, it can be seen that as the content of the oxidant increases, the size increases. It appeared from 62.7 (±22.1) nm to 273.7 (±27) nm distributions, and it was confirmed that when the concentration of the oxidant is 80 wt %, it grew up the most. This is because PEDOT is synthesized as the in situ synthesis proceeds by anionic polymerization, and the particles are formed more actively as expected. An increase in the molecular weight is expected, but the experimental analysis is limited, so it can only be seen by analogy with each particle size.

**Figure 4:**
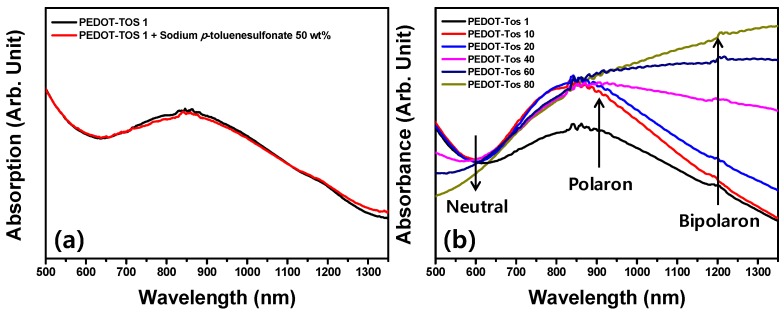
Ultraviolet-visible-near-infrared (UV-Vis-NIR) absorption spectra of PEDOT-Tos (1 mg·mL^−1^ dispersed in *n*-butanol) are plotted. (**a**) PEDOT-Tos 1 synthesized with a small concentration of the oxidant (1 wt %), which cannot be doped more than the intrinsic level by excess sulfonate (sodium *p*-toluenesulfonate). (**b**) Peaks increasing with the concentration of the oxidant used for the synthesis of PEDOT-Tos are observed at approximately 1200 nm (bipolaron state) in the absorption spectra.

Further, we investigated whether the carrier concentration can be modified by the addition of the excess tosylate. We added 50 wt % of sodium *p*-toluenesulfonate solution in *n*-butanol to the synthesized PEDOT-Tos 1 to confirm whether the additional sulfonate can change the doping level of PEDOT-Tos. The ultraviolet-visible-near-infrared (UV-Vis-NIR) spectra in Figure 4a show absorptions of PEDOT-Tos 1 and PEDOT-Tos 1 with the sodium *p*-toluenesulfonate solution. There is almost no difference between these two spectra, indicating that the sample cannot be doped further with the excess sulfonates. On the other hand, the absorption peaks at large wavelengths (~1200 nm), which indicate the bipolaron state of the doped PEDOT-Tos [26,34], gradually increase with the concentration of the oxidant used for the synthesis of PEDOT-Tos, as shown in Figure 4b. This is because the Ferric cation (Fe^3+^) from the oxidant accelerates the polymerization of the PEDOT-Tos from the monomer (EDOT) while the negatively charged sulfonate acts as a counter-ion stabilizing a highly doped bipolaron state. The Ferric cation definitely plays an important role to polymerize the EDOT to the PEDOT-Tos. Peaks around at 600 nm indicated the neutral state, and about at 900 nm indicated the polaron state of the synthesized PEDOT-Tos. As oxidation progresses much more, the benzoid structure (conjugated state) is formed more than the quinoid structure (de-doping state). Accordingly, the conjugated length finally becomes long enough, so that the peaks around 1200 nm appear to increase as shown in Figure 4b.

The particle sizes of PEDOT-Tos gradually increase with the concentration of the oxidant; the larger PEDOT exhibits more bipolaron states. It may possible by providing pathways for the generated carriers in PEDOT chains.

**Figure 5:**
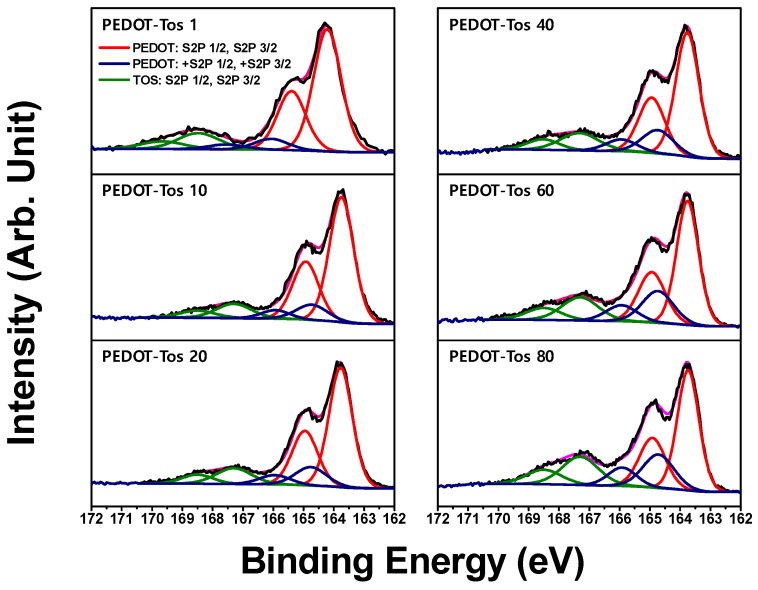
X-ray photoelectron spectroscopy (XPS) analysis confirms the ratio of sulfonate anions (Tos) and thiophene (EDOT) tend to increase with the concentrations of the oxidant. The doublet with a binding energy of about from 166 to 170 eV represents Tos, whereas the thiopehen of the PEDOT chain has a binding energy of about 162.5 to 166.5 eV. The PEDOT-Tos 1 exhibits peak shifts toward higher energy, which is considered to be a weak interaction with the counter-ion.

In order to investigate the factors affecting the different doping states (oxidation level) of PEDOT-Tos, we analyzed the molar ratio between the PEDOT chain and counter-ion (tosylate, Tos) by XPS. In general, the ratio of counter-ion and PEDOT content was calculated and expressed through the analysis of S (*2p*) orbital through XPS analysis as a method of quantifying the doping state or the oxidation state of PEDOT [35]. In order to quantitatively analyze the oxidation state, the XPS analysis was carried out as shown in Figure 5, and it was found that the oxidation state was increased up to a maximum of 18.9% depending on the concentration of the oxidant. The XPS signals S (*2p*) from 162.5 to 166.5 eV indicate the binding energy of thiophene units in PEDOT, and the binding energy of the tosylate is determined by the XPS signals from 166 to 170 eV. In general, the ratio of a counter-ion to thiophene is 1:4 (25%). However, the percentage progressed in this evaluation by XPS analysis indicates a maximum of 18.9% that represents a low oxidation level, so the electrical conductivity was measured lower (0.96 S·cm^−1^) than known (about 300 S·cm^−1^) [19]. Additionally, the reason why the electric conductivity is low can be explained by the fact that the above-mentioned synthesis was performed without a base agent such as pyridine. This is consistent with the results of a previous published paper [26]. In this study, it is aimed to observe the change of the characteristics of thermoelectric material from the change of the oxidant concentration. Therefore, if the optimum conditions are found by this method, and synthesized in a basic atmosphere such as pyridine, it will be better strategy to get an advanced performance.

PEDOT-Tos 1 was synthesized at a very low oxidant concentration (1 wt %), which shows a different peak shift about 0.6 eV higher than that synthesized at other oxidant concentrations as shown in Figure 5. As it is considered that the interaction formed between PEDOT and Tos formed is weak, but the oxidation level seems to be slightly higher than the trend of oxidant concentration. This could be because the polymer chain formation of the PEDOT was not formed properly at a very low concentration of the oxidant. Accordingly, the conjugated length is far too short due to the formation of quinoid structure (de-doping state) rather than the benzoid structure (conjugated state). Based on Hückel’s rule, it can be also assumed that the shorter chain length is formed, the larger band gap (HOMO (Highest Occupied Molecular Orbital) and LUMO (Lowest Unoccupied Molecular Orbital) is generated, which results in the lower electrical properties.

**Figure 6:**
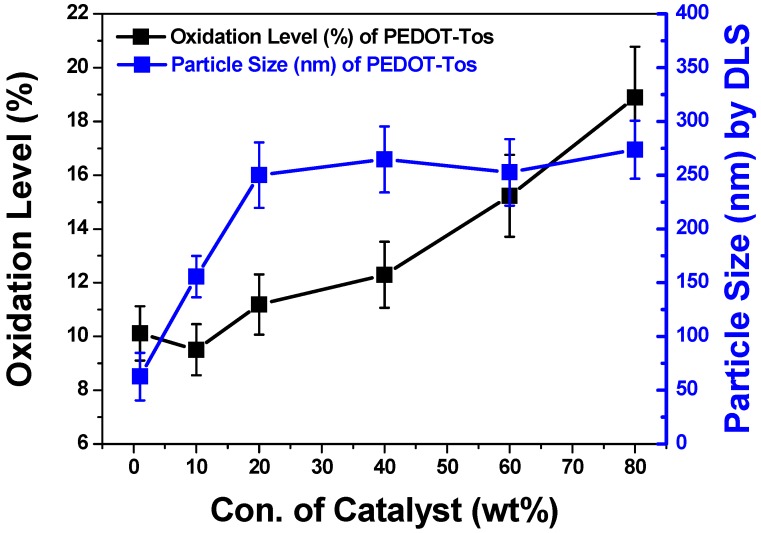
The oxidation level (%) by XPS analysis and the particle size (nm) by DLS analysis are plotted with the concentrations of the oxidants. Both properties tend to increase with loading the oxidant.

Figure 6 shows that plotting the changes in the oxidation level and particle size from the XPS and the DLS, respectively, depending on the oxidant concentration. In other words, it generally shows the potential to control the oxidation level by changing the concentration of the oxidant. This indicates that even if no reducing agent is used, the desired oxidation level can be achieved by properly controlling the concentration of the oxidant.

**Figure 7:**
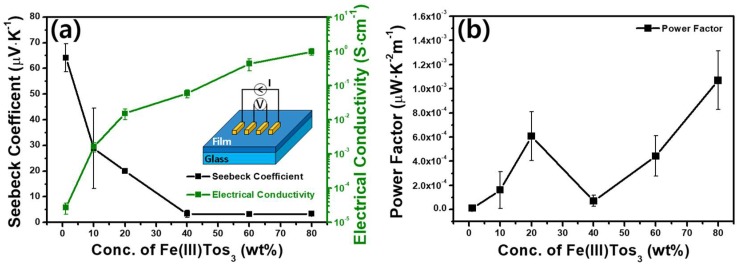
(**a**) The electrical conductivities and the Seebeck coefficients of PEDOT-Tos with different catalyst concentrations (the error bars are based on the standard deviations of the measured data) are plotted (the error bars are based on the standard deviations of the measured data). (**b**) Power factor (S^2^·σ) based on the measured data is also calculated with µW·K^-2^m^-1^ unit and plotted.

The electrical conductivity of PEDOT-Tos increases with the concentration of the oxidant, while the Seebeck coefficient decreases, as shown in Figure 7a. The catalysts were removed by washing the polymers with *n*-butanol after the synthesis; the remaining Fe content was under 3 ppm in all of the synthesized PEDOT-Tos, as confirmed by atomic absorption spectroscopy. However, the amount of remaining catalyst was sufficiently small to be ignored; it does not affect the electrical properties of PEDOT-Tos. The highest electrical conductivity measured from the samples was approximately 0.96 S·cm^-1^. The values were lower than those of PEDOT:PSS processed with additives and PEDOT-Tos polymerized using a polymer template. As described previously, it is due to the absence of the inhibitor such as pyridine. The Seebeck coefficient changed from 3.3 to 64.1 µV·K^−1^ with the variations in the particle size and saturated doping level. The lowest electrical conductivity was approximately 2.7 × 10^−5^ S·cm^−1^, while the highest Seebeck coefficient was approximately 64.1 µV·K^−1^. The origin of the opposite behaviors of the electrical conductivity and Seebeck coefficient can be attributed to the Fermi level approaching the carrier transport state, leading to an increase in the electrical conductivity and decrease in the Seebeck coefficient. The tradeoff behavior of these two properties is because the high conductivity can only be possible with heavily doped level, where the charge-transport energy level comes to close to the Fermi level [36]. This phenomenon could be defined as following “Boltzman equation” by Fritzsche [37].

(2) $$S=\frac{k}{e}\left[\frac{\left(E_{F}-E_{T}\right)}{kT}+A\right]$$

where *k* is the Boltzmann constant, *e* is the electronic charge, *E_F_* and *E_T_* are the Fermi energy level and the transport energy level, respectively. The term “*A*” means the temperature independence of the heat of the transport constant.

Figure 7b shows the resulting power factor (S^2^·σ). Although there is a slight fluctuation depending on the conductivity and the Seebeck coefficient, and the maximum value is very low, and was 1.07 × 10^−3^ µW·K^−2^m^−1^**.** The point of this work is that it presents a methodological possibility rather than high thermopower results.

## 4. Conclusions

In conclusion, the in situ polymerization and doping of PEDOT-Tos with various concentrations of iron(III) *p*-toluenesulfonate hexahydrate was carried out to modify the oxidation levels of the conjugated polymer, PEDOT. Besides, the particle size also became larger with loading the oxidant. As expected, the ratio of the tosylate to the thiophenes presented an increase from 9.5% to 18.9% in the oxidation level with the concentration of the oxidant. In addition, as the concentration of the oxidant increased, the size of PEDOT-Tos increased from 62.7 (±22.1) nm to 273.7 (±27) nm as a result of DLS analysis. Thus, the concentrations of the oxidant affect the electrical conductivity and the Seebeck coefficient. The number of generated bipolaron states induced by the PEDOT-Tos increased with the concentration of the oxidant, leading to an increase in the electrical conductivity from about 2.7 × 10^−5^ to 0.96 S·cm^−^^1^, whereas the thermopower decreased with the doping. We investigated the doping behavior of PEDOT-Tos depending on the concentrations of the oxidant. The electrical conductivity could be widely varied as well as the Seebeck coefficient. It is assumed that low performance is due to the fact that the amount of pyridine is excluded from the experimental parameters. This study provides a simpler alternative route than the use of a reducing agent to optimize the power factor of PEDOT. This reveals a novel approach to modify the power factors of conjugated polymers by modifying just the concentration of the oxidant. In addition, if we use pyridine or weak base oxidant with the results in this work, we would achieve a higher power factor.
